# Two novel in vitro assays to screen chemicals for their capacity to inhibit thyroid hormone transmembrane transporter proteins OATP1C1 and OAT4

**DOI:** 10.1007/s00204-024-03787-2

**Published:** 2024-05-18

**Authors:** Fabian Wagenaars, Peter Cenijn, Zhongli Chen, Marcel Meima, Martin Scholze, Timo Hamers

**Affiliations:** 1 Amsterdam Institute for Life and Environment (A-Life), Vrije Universiteit Amsterdam (VU), De Boelelaan 1085, 1081 HV Amsterdam, The Netherlands; 2 Academic Centre for Thyroid Diseases, Department of Internal Medicine, Erasmus University Medical Center Rotterdam, 3015 GD Rotterdam, The Netherlands; 3 Centre for Pollution Research and Policy, College of Health, Medicine and Life Sciences, Brunel University London, Kingston Lane, Uxbridge, UB8 3PH UK

**Keywords:** Endocrine disrupting chemicals, Thyroid hormones, In vitro bioassays, Organic anion transporter polypeptide 1C1, Organic anion transporter 4

## Abstract

Early brain development depends on adequate transport of thyroid hormones (THs) from the maternal circulation to the fetus. To reach the fetal brain, THs have to cross several physiological barriers, including the placenta, blood–brain-barrier and blood–cerebrospinal fluid-barrier. Transport across these barriers is facilitated by thyroid hormone transmembrane transporters (THTMTs). Some endocrine disrupting chemicals (EDCs) can interfere with the transport of THs by THTMTs. To screen chemicals for their capacity to disrupt THTMT facilitated TH transport, in vitro screening assays are required. In this study, we developed assays for two THTMTs, organic anion transporter polypeptide 1C1 (OATP1C1) and organic anion transporter 4 (OAT4), both known to play a role in the transport of THs across barriers. We used overexpressing cell models for both OATP1C1 and OAT4, which showed an increased uptake of radiolabeled T4 compared to control cell lines. Using these models, we screened various reference and environmental chemicals for their ability to inhibit T4 uptake by OATP1C1 and OAT4. Tetrabromobisphenol A (TBBPA) was identified as an OATP1C1 inhibitor, more potent than any of the reference chemicals tested. Additionally perfluorooctanesulfonic acid (PFOS), perfluoroctanic acid (PFOA), pentachlorophenol and quercetin were identified as OATP1C1 inhibitors in a similar range of potency to the reference chemicals tested. Bromosulfophthalein, TBBPA, PFOA and PFOS were identified as potent OAT4 inhibitors. These results demonstrate that EDCs commonly found in our environment can disrupt TH transport by THTMTs, and contribute to the identification of molecular mechanisms underlying TH system disruption chemicals.

**Supplementary Information:**

The online version contains supplementary material available at 10.1007/s00204-024-03787-2.

## Introduction

Early neurodevelopmental processes are heavily dependent on adequate amount of thyroid hormones (THs) in the fetal brain. Severe hypothyroidism and hyperthyroidism during fetal brain development cause irreversible structural alterations to the fetal brain which lead to cognitive defects, motor function loss and behavioral problems. TH concentrations in the brain are kept within a small optimal range, as even modest changes in TH homeostasis can lead to loss of IQ (Korevaar et al. 2018). Most of the TH in the brain is derived from circulating thyroxine (T4), which gets locally converted by deiodinase enzymes into biologically active triiodothyronine (T3). The fetal thyroid gland does not produce a sufficient amount of TH during the initial stages of gestation, therefore the fetus is dependent on a maternal supply of TH (Chan and Kilby 2000). To reach the fetal brain, THs need to cross several physiological barriers: the placenta, blood–brain-barrier (BBB) and the blood–cerebrospinal fluid barrier (BCSFB). Transport across these barriers, as well as cellular influx and efflux, is mediated through TH transmembrane transporters (THTMTs). Several THTMTs from different protein families have been identified to date. Monocarboxylate 8 (MCT8) is the best studied THTMT and mutations of MCT8 are linked with a severe neurodevelopment disorder called the Allan–Herndon–Dudley syndrome (AHDS) (Schwartz et al. 2005). Other THTMTs than MCT8 are also considered to play a major role in TH transport, however the full range of important THTMTs is not yet known.

Organic anion transporter polypeptide 1C1 (OATP1C1) is another THTMT that plays an important role in the transport of T4 across the BBB and BCSFB. OATP1C1 has been characterized as a bidirectional high affinity T4 transporter independent of sodium and adenosine triphosphate (Pizzagalli et al. 2002). OATP1C1 is abundantly present in astrocytes, microglia cells, pyramidal and interneurons in the motor cortex and the apical and basolateral surface of the choroid plexus epithelial cells in humans (Roberts et al. 2008; Wang et al. 2023). OATP1C1 is also highly expressed in rodent cortical microvessels, but not in humans or macaques (Roberts et al. 2008; Wang et al. 2023). The importance of OATP1C1 as a THTMT is further elucidated in animal models. In zebrafish, knockout of OATP1C1 results in structural alteration of radial glial cells, shorter axon neurons, enlarged thyroid gland and changes in behavioral locomotor activity (Admati et al. 2020). In rodent models, knock out of OATP1C1 leads to decreased TH in the brain, without abnormal circulating THs, and hypothyroidism of the central nervous system, but lacks neurological abnormalities and behavioral problems typically observed in human hypothyroid patients (Mayerl et al. 2012). However, rodents lacking both OATP1C1 and MCT8 do exhibit severe neurodevelopmental adverse effects comparable to effects seen in human MCT8-deficiency patients. Interestingly, MCT8 knockout rodent models alone do not display these neurological effects, suggesting that OATP1C1 provides an alternative route of TH transport in this case. To date, only a single case study of OATP1C1 deficiency has been reported in humans, detailing a mutation in the gene encoding OATP1C1 that led to brain hypothyroidism due to reduced T4 transport capabilities of OATP1C1 (Strømme et al. 2018). This patient exhibited symptoms comparable to hypothyroidism, such as cognitive degeneration, loss of motor functions and intolerance to cold. However, serum TH concentrations, for both T3 and T4, were within a normal range. Overall, these data indicate that OATP1C1 is a major contributor to TH homeostasis in the brain by facilitating T4 transport across the BBB and BCSFB.

In the placenta, a substantial amount of T4 transport still cannot be attributed to known THTMTs, including MCT8 (Loubière et al. 2012). A good candidate for this unidentified T4 placental THTMT is organic anion transporter 4 (OAT4). OAT4 is highly expressed in the basal membrane of the syncytiotrophoblasts in the placenta (Cha et al. 2000) and is a known important transporter in the placenta, where it can transport sulfated steroid hormone precursors, perfluorocarboxylates and other organic anions (Cha et al. 2000; Kummu et al. 2015; Tomi et al. 2015). Recently, OAT4 has been identified as a novel efficient T4 transporter (Chen et al. 2023). As such there is increasing evidence that OAT4 is an important THTMT in the placenta.

Exposure to endocrine disrupting chemicals (EDCs) can potentially interfere with the transport of TH across the physiological barriers and cause altered TH concentrations in the fetal brain. Therefore, assays that can detect chemical interferences on TH transport are of vital importance. Particularly, there is a need for rapid in vitro screening assays that can identify potential EDCs acting upon THTMT mediated transport. Recently such screening in vitro assay has been described for MCT8 (Jayarama-Naidu et al. 2015; Wagenaars et al. 2023), but no assays exist for other THTMTs, such as OATP1C1 and OAT4. In this study we developed in vitro assays that can identify potential EDCs that interfere with OATP1C1 and OAT4 mediated TH transmembrane transport, by generating THTMT overexpressing cell models and exposing them to radioactive labelled T4. These assays were subsequently used in inhibition studies to screen a set of environmental contaminants.

## Materials and methods

### Materials

Indocyanine green (ICG) was purchased from Cayman Chemical (Uden, The Netherlands). perfluorooctanoic acid (PFOA) was obtained from Acros Organics (Landsmeer, the Netherlands). Diclofenac (DIC), bisphenol-F (BPF), perfluorooctanesulfonic acid (PFOS) and imidacloprid (IMI) were obtained from Fluka (Geel, Belgium). Bromosulfophthalein (BSP) was purchased from Alfa Aesar (Kandel, Germany). Bisphenol-S (BPS) and bisphenol-Z (BPZ) were obtained from TRC Canada (Toronto, Canada). Sulforhodamine 101 (SR101), lesinurad (LESI), quercetin (QCT), bisphenol-AF (BPAF), triclosan (TCS), 4-hydroxycinnamic acid (4HCA) were all obtained from Merck Life Science. All other chemicals were obtained from Sigma Aldrich (Zwijndrecht, the Netherlands). l-Tryptophan was dissolved in uptake buffer and BCH in 1 M NH4OH, while all other chemicals were dissolved in dimethylsulfoxide (DMSO). ^125^I-T4 was purchased from Perkin Elmer (Groningen, the Netherlands). Plastics used during the experiments were purchased from Starstedt (Etten-Leur, The Netherlands).

### Generation of OAT4 construct

To generate Madin-Darby canine kidney (MDCK-1) cells that stably express OAT4, the gene insert of OAT4 from the pCMV6-OAT4-myc-DDK vector (Chen et al. 2023) was amplified using specific primers (forward: 5′-CACCATGGCGTTCTCGAAGCTCTT-3′; reverse: 5′-GAGCGAGGTACTTTCCACAGTGA-3′) and then subcloned into the pENTR™/D-TOPO® Vector using the pENTR™/D-TOPO™ Cloning Kit (Thermo Fisher). Subsequently OAT4 in pENTR™/D-TOPO® Vector was shuffled into the pLenti6/V5-DEST vector (Thermo Fisher) using Gateway™ LR Clonase™ II Enzyme mix (Thermo Fisher) according to the manufacture’s protocol.

### Generation of stable MDCK-1 cells over-expressing OAT4 and OATP1C1

To generate an OAT4 lentivirus, 293FT cells were transfected with 3 µg pLenti6/V5-DEST-OAT4 or empty pLenti6/V5-DEST vector, the packaging vectors (3 µg pLP1and 3 µg pLP2) and 3 µg envelope vector pVSV-G at 70% confluency using X-tremeGENE 9 Transfection Reagent (Roche). The cell culture medium was refreshed 24 h after transfection and viral supernatants were collected at 48 h and 72 h post transfection and filtered through a 0.45 µM PES filter (GE Healthcare). To generate an OAT4 stable cell line, MDCK-1 cells were plated in 12-well plates with full growth medium and transduced with fresh viral supernatants in the absence of antibiotics in medium. After 2 days, MDCK-1 cells transduced with OAT4 (MDCK-OAT4) or empty vector (MDCK-DEST) were selected in full growth medium supplemented with 2 µg/mL blasticidin and further maintained under blasticidin selection. Chinese hamster ovary (CHO-K1) cells overexpressing OATP1C1 (CHO-K1 OATP1C1) were generated as previously described by Pizzagalli et al. (2002) and kindly provided by the University Hospital of Zürich (Dr Michele Visentin).

### Cell culture and seeding

MDCK-OAT4 and MDCK-DEST cells were cultured in DMEM:F12 (l-glutamine, 15 mM HEPES) (Gibco) supplemented with (100 µg/mL penicillin + 100 µg/mL streptomycin), 10% fetal calf serum (Gibco) and 2 µg/mL blasticidin (Sigma). CHO-K1 OATP1C1 or WT cells were cultured in F12 nutrient mix (Gibco) supplemented with (100 µg/mL penicillin + 100 µg/mL streptomycin), 10% fetal calf serum (Gibco) and 100 µg/µL G418 (Sigma). All cell cultures were incubated at 37 °C with 5% CO_2_ saturation until 80% confluency. MDCK-OAT4 and MDCK-DEST cells were seeded in 96 wells plate at a density of 4 × 10^5^ cells in 100 µL medium per well and incubated for 24 h before the start of the MDCK-OAT4 TH uptake assay. CHO-K1 OATP1C1 and WT cells were seeded in 96 wells plate at a density of 3 × 10^5^ cells in 100 µL medium per well 72 h before the CHO-K1 OATP1C1 TH uptake assay. Medium from CHO-K1 OATP1C1 or WT assay plates was changed to serum-free F12 medium containing 5 mM sodium-butyrate 24 h before start of the CHO-K1 OATP1C1 TH uptake assay to increase transfected expression of SV-40 promotor plasmids.

### ***THTMT TH uptake assay with ***^***125***^***I-T4 tracer***

To start the THTMT assay with ^125^I-T4 tracer, medium was removed from assay plates and cells were washed with 200 µL phosphate buffered saline (PBS). Each well received 80 µL of uptake buffer (125 mM NaCl, 10 mM HEPES, 5 mM KCl, 1.3 mM CaCl_2_, 1.2 mM MgCl_2_, pH adjusted to 7.4 and freshly added 5.6 mM d-glucose). Subsequently 10 µL of a tenfold stock of test compound in uptake buffer (solvent concentration 1% v/v) was added to each well. The assay was started with the addition of 10 µL of T4 tracer solution, with a final concentration of 10 nM cold T4 and approximately 10.000 counts per minute (cpm) ^125^I-T4, solved in uptake buffer. Cells were exposed at 37 °C for 20 min (MDCK-OAT4) or 30 min (CHO-K1 OATP1C1), unless stated differently. After exposure, cells were immediately washed with ice-cold PBS supplemented with 0.1% bovine serum albumin (BSA). Subsequently, cells were lysed in 50 µL of 0.1 M NaOH under continuous shaking at room temperature. After 20 min, cell lysates were manually transferred into separate 5 mL polypropylene tubes. One tube was also prepared with 10 µL of the T4 tracer solution. Radioactivity was measured in a Wizard^2^ gamma counter (Perkin Elmer).

For the CHO-K1 OATP1C1 TH uptake assay total of 29 chemicals (Table 1) were selected for the EDC screening based on available literature data. Two negative control compounds (IBU, BCH) and five positive control chemicals (DIC, PRB, ICG, SR101, BSP, LESI) were included. The same chemicals were selected for the MDCK-OAT4 TH uptake assay, with the exception of BPF, VRP and TCS. Two positive control chemicals (PRB, LESI) were included, while no available literature have yet described negative control chemicals for OAT4 mediated TH uptake. The THTMT TH uptake assay was then performed as described earlier. A range finding study was performed at either 10 or 100 µM, depending on solubility of the chemical. A chemical that significantly inhibited T4 uptake by < 80% of the control was considered as a positive hit, i.e. inhibitors (One Way ANOVA). A concentration of 100 µM ICG was used as a reference chemical for OATP1C1 and 100 µM LESI for OAT4. At least three independent experiments were performed for each chemical. Each individual experiment contained three technical replicates of each control and concentration tested.

**Table 1:** Test compounds and their inclusion criteria

CAS	Compound	Abbreviation	Inclusion criteria	Reference	Supplier
15687-27-1	Ibuprofen	IBU	Negative control (OATP1C1)	Westholm et al. (2009)	Sigma-Aldrich
20448-79-7	2-Amino-bicycloheptane-2-carboxylic acid	BCH	Negative control (OATP1C1)	Tohyama et al. (2004)	Sigma-Aldrich
15307-79-6	Diclofenac	DIC	OATP1C1 inhibitor	Westholm et al. (2009)	Fluka
57-66-9	Probenecid	PRB	OATP1C1/OAT4 inhibitor	Tohyama et al. (2004); Chen et al. (2023)	Sigma-Aldrich
3599-32-4	Indocyanine green	ICG	OATP1C1 inhibitor	Westholm et al. (2009)	Cayman Chemical
60311-02-6	Sulforhodamine 101	SR101	OATP1C1 inhibitor	Bakos et al. (2020)	Merck Life Science
71-67-0	Bromosulfophthalein	BSP	OATP1C1 substrate	Sugiyama et al. (2003)	Alfa Aesar
878672-00-5	Lesinurad	LESI	OAT4 inhibitor	Chen et al. (2023)	Merck Life Science
51-52-5	Propylthiouracil	PTU	TPO inhibitor	Friedman et al. (2016)	Sigma-Aldrich
117-39-5	Quercetin	QCT	TPO inhibitor	Friedman et al. (2016)	Merck Life Science
1763-23-1	Perfluorooctanesulfonic acid	PFOS	NIS inhibitor/TTR binding	Buckalew et al. (2020); Weiss et al. (2015)	Fluka
73-22-3	l-Tryptophan	TRP	LAT1/2 inhibitor	Chen et al. (2022a, b)	Sigma-Aldrich
13292-46-1	Rifampicin	RIF	OATP inhibitor	Chen et al. (2022a, b)	Sigma-Aldrich
152-11-4	Verapamil	VRP	OATP inhibitor	Chen et al. (2022a, b)	Sigma-Aldrich
335-67-1	Perfluorooctanoic acid	PFOA	TTR binding	Weiss et al. (2009)	Sigma-Aldrich
87-86-5	Pentachlorophenol	PCP	TTR binding	Hamers et al. (2006)	Sigma-Aldrich
79-94-7	Tetrabromobisphenol-A	TBBPA	TTR binding	Hamers et al. (2006)	Sigma-Aldrich
115-09-3	Methyl mercury	MeHg	MCT8 inhibitor	Wagenaars et al. (2023)	Sigma-Aldrich
33889-69-9	Silychristin	SY	MCT8-inhibitor	Johannes et al. (2016)	Merck Life Science
80-05-7	Bisphenol-A	BPA	MCT8-inhibitor	Wagenaars et al. (2023)	Sigma-Aldrich
843-55-0	Bisphenol-Z	BPZ	MCT8-inhibitor	Wagenaars et al. (2023)	TRC Canada
1478-61-1	Bisphenol-AF	BPAF	MCT8-inhibitor	Wagenaars et al. (2023)	Merck Life Science
80-09-1	Bisphenol-S	BPS	BPA-like		TRC Canada
620-92-8	Bisphenol-F	BPF	BPA-like		Fluka
3380-34-5	Triclosan	TCS	decreased brain T4 in pups	Gilbert et al. (2021)	Merck Life Science
138261-41-3	Imidacloprid	IMI	decreased plasma T4 in rats	Ibrahim et al. (2015)	Fluka
4999-79-5	β-Estradiol-3-sulfate	β-E3S	OAT4 substrate	Skwara et al. (2017)	Sigma-Aldrich
69-93-2	Uric acid	UA	OAT4 substrate	Skwara et al. (2017)	Sigma-Aldrich
7400-08-0	4-Hydroxycinnamic acid	4HCA	OAT4 substrate	Wong et al. (2011)	Merck Life Science

### Cell viability assay

Cell viability was assessed through the quantitation of ATP present in cells using the commercially available CellTiter-Glo® luminescent cell viability assay and performed according to the manufacturer’s instruction (Promega, Leiden, the Netherlands). Briefly, cells were seeded as described earlier in opaque-walled clear-bottom 96 well plates. On the day of the experiment, each well was washed with 200 µL phosphate buffered saline (PBS) and exposed to 80 µL of uptake buffer, 10 µL of a tenfold stock of test compound solved in uptake buffer or control and 10 µL of T4 (final concentration 10 nM). 0.1% DMSO was used as a solvent control, while 10% DMSO was used as a positive control. After exposure for the indicated times similar to THTMT TH uptake assay, cells were immediately washed with ice-cold PBS + 0.1%BSA and exposed to 100 µL of the CellTiter-Glo® Reagent. Plates were then shaken on an orbital shaker for 2 min and left to incubate at room temperature for 10 min. Luminescence was measured on a BioTek Cytation 5 microplate reader. A similar range finding study was performed as for the THTMT TH uptake assay, where chemicals that showed a decrease in cell viability < 90% of the control were subsequently tested in eight concentrations to obtain a full concentration response curve. Each individual experiment was repeated at least twice and contained three technical replicates of each control and concentration tested.

## Data analysis

Raw values were normalized to the total amount of radioactivity in the T4 tracer solution. These values were expressed as a percentage of total radioactivity added (or uptake percentage) and used for all further data analysis. Statistically significant differences between the T4 uptake of transfected and wild type cells, in the presence or absence of inhibitor, were determined by two-way ANOVA (α = 5%, two-sided). Concentration–response analysis for the T4 uptake in both cell models was performed using a best-fit-approach as previously described (Scholze et al. 2001; Wagenaars et al. 2023). In brief, uptake values were normalized to the median response of the vehicle and positive controls for each plate. A control renormalization step was performed on a plate-to-plate basis as previously described (Krebs et al. 2018; Wagenaars et al. 2023). Chemical-specific potencies were derived from the regression analysis on the control-normalized effect responses and expressed as benchmark concentration (BMC), with the benchmark response level (BMR) determined as 80% of the vehicle control (i.e. 20% inhibition). Statistical analysis were performed using GraphPad Prism version 8 for Windows (GraphPad Software, San Diego, California USA) and SAS 9.3 (SAS Institute, Cary NC).

## Results

### TH uptake in CHO-K1 OATP1C1 and MDCK-OAT4

**Fig. 1:**
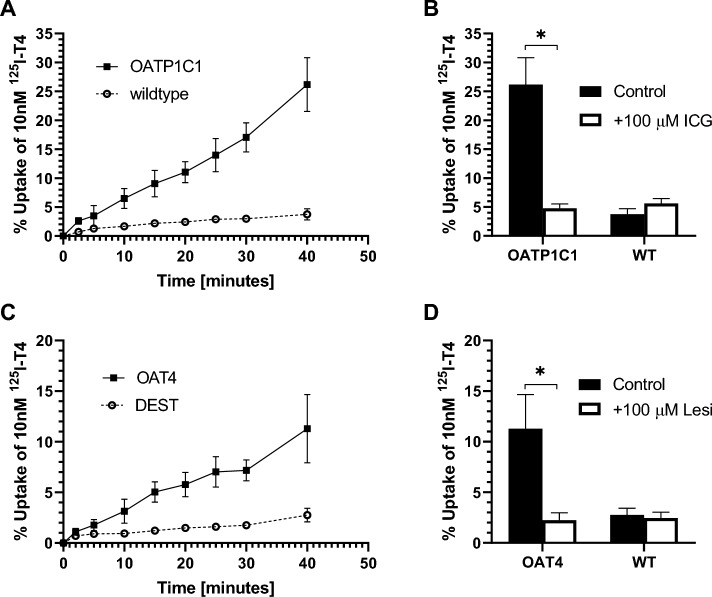
Kinetics of the CHO-K1 OATP1C1 and MDCK-OAT4 T4 uptake assay. **A** The uptake of ^125^I-T4 in time, expressed as a percentage of total T4, in CHO-K1 OATP1C1 or CHO-K1 wildtype cells. **B** Comparison between the percentage of ^125^I-T4 uptake in CHO-K1 OATP1C1 and CHO-K1 WT cells after 40 min incubation, in the absence and presence of 100 µM ICG. **C** Uptake of ^125^I-T4 uptake in time expressed as a percentage of total T4 in MDCK-OAT4 and MDCK-DEST cells over time. **D** Comparison of the percentage of ^125^I-T4 uptake in MDCK-OAT4 and MDCK-DEST cells after 40 min incubation, with and without 100 µM LESI. Data is shown as the mean ± SD, N = 3. Comparisons with a p values below 0.05 are indicated by a star

To characterize the in vitro kinetics, the TH uptake in CHO-K1 OATP1C1 and MDCK-OAT4 was measured at different incubation times. In CHO-K1 OATP1C1 cells, T4 uptake increased over time up to ~ 25% T4 added at t = 40 min (Fig. 1A), compared to < 4% in CHO-K1 WT cells. The ~ sixfold increase in T4 uptake in CHO-K1 OATP1C1 cells compared to CHO-K1 wildtype cells at t = 40 min decreased back to control when co-exposed to a known inhibitor of OATP1C1, i.e. 100 µM ICG (Fig. 1B). To keep in the linear T4 uptake range, all further experiments were performed with 30 min incubation times, where T4 uptake in CHO-K1 OATP1C1 (~ 17%) was 5.5-fold higher than CHO-K1 wildtype cells (~ 3%). T4 uptake in MDCK-OAT4 cells increased up until t = 25 min, plateaued at t = 30 min at ~ 7% T4 uptake (Fig. 1C). T4 uptake in MDCK-DEST cells increased up until t = 40 min at ~ 4% T4 uptake. At t = 40 min, MDCK-OAT4 cells had a fivefold higher T4 uptake compared to MDCK-DEST cells. Similar as for the CHO-K1 OATP1C1 cells, T4 uptake by the MDCK-OAT4 cells decreased back to control concentrations when cells were co-exposed to a known OAT4 inhibitor, i.e. 100 µM LESI (Fig. 1D). For all experiments hereafter an incubation time of 20 min was used, where T4 uptake in MDCK-OAT4 (~ 5.7%) was ~ fourfold higher compared to MDKC-DEST cells (~ 1.4%).

### EDC screening with THTMT TH uptake assays

**Fig. 2:**
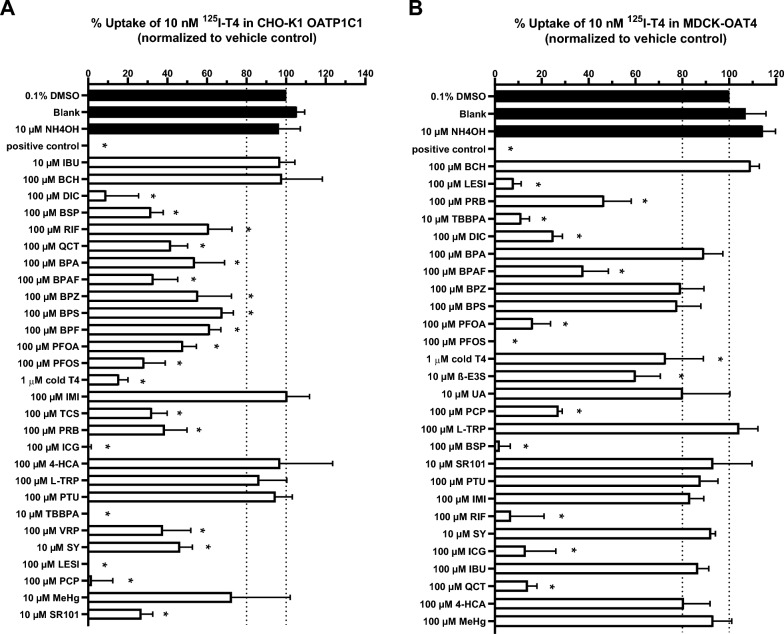
Percentage of ^125^I-T4 uptake compared to the vehicle control 0.1% DMSO in **A** CHO-K1 OATP1C1 and **B** MDCK-OAT4 cells in the presence of selected chemicals. CHO-K1 OATP1C1 and MDCK-OAT4 cells were exposed to 10 nM T4 and 10–100 µM of selected chemicals for 30 min and 20 min respectively. Chemicals that decreased T4 uptake below 80% (i.e. 20% inhibition) after exposure were considered positive hits. 0.1% DMSO was used as vehicle control, 100 µM ICG was used as a positive control for OATP1C1 and 100 µM LESI for OAT4 (in black). Results are shown as mean ± SD, N = 3. Comparisons with a p values below 0.05 are indicated by a star

To screen for chemicals that inhibit the THTMTs, all selected chemicals were initially tested at a single high concentration of 100 or 10 µM, depending on their solubility (Fig. 2). There was no difference between T4 uptake exposed to solvent controls 0.1% DMSO or 10 µM NH_4_OH and the uptake buffer (blank). In the CHO-K1 OATP1C1 TH uptake assay, both negative control compounds, IBU and BCH, did not decrease T4 uptake (Fig. 2A). Neither IMI, 4HCA, L-TRP, PTU nor MeHg decreased OATP1C1 mediated T4 uptake significantly below 80%, while all other compounds did. Highest inhibition was found for TBBPA followed by (in decreasing order of potency) ICG, LESI, PCP, PFOS, BSP, TCS, PRB, BPAF, DIC, QCT, SR101, PFOA, BPA, BPZ, VRP, SY, RIF, BPF and BPS. Addition of an excess of unlabeled 1 µM T4 also quenched the uptake of ^125^I-T4 in CHO-K1 OATP1C1 cells, indicating a saturable uptake (Fig. 2A). In the MDCK-OAT4 TH uptake assay, no decrease in T4 uptake below 80% was observed for BCH, BPA, BPZ, BPS, UA, L-TRP, SR101, PTU, IMI, SY, IBU, 4HCA and MeHg (Fig. 2B), while all other chemicals did. Highest inhibition was found for PFOS, followed by (in decreasing order of potency) BSP, LESI, RIF, TBBPA, ICG, QCT, PFOA, DIC, PCP, BPAF, PRB, TCS, VRP, β-E3S and BPF.

**Fig. 3:**
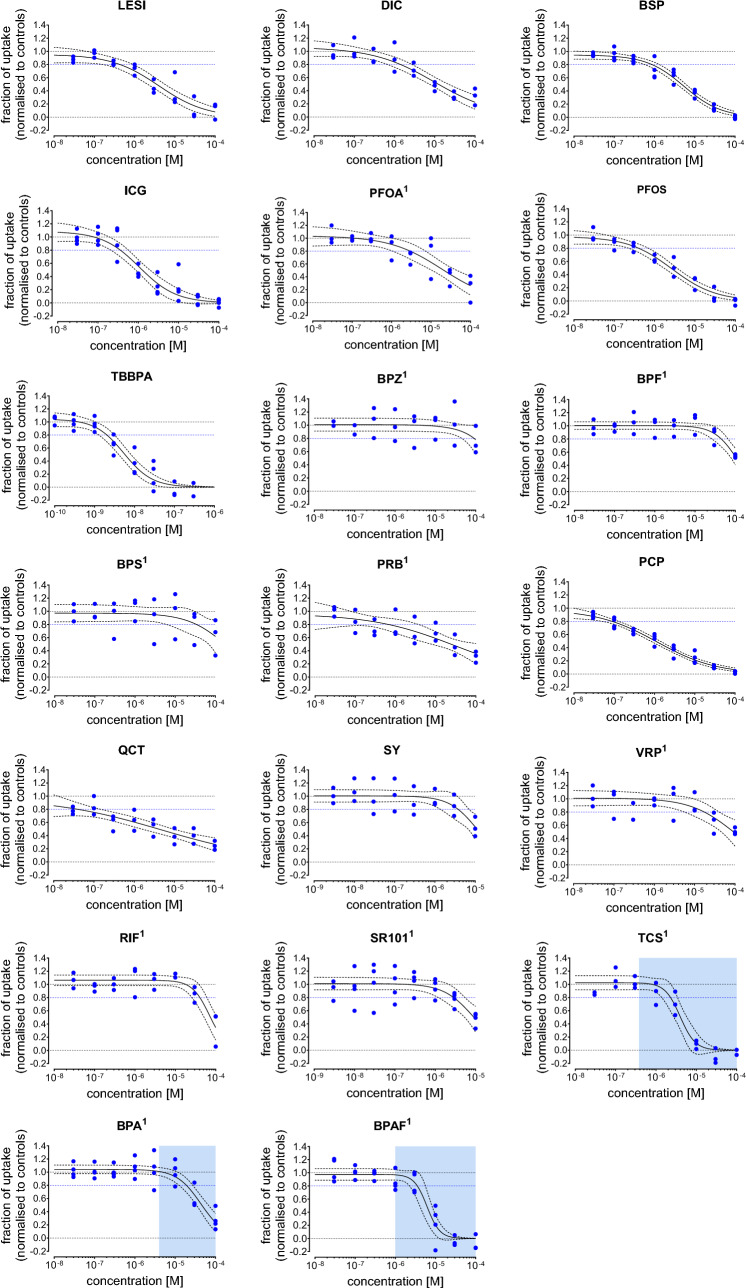
Concentration–response curves for test chemicals that showed inhibition towards OATP1C1 mediated uptake of T4 (10 nM) in the CHO-K1 OATP1C1 TH uptake assay. The fraction of ^125^I-T4 uptake is normalized to the positive control, 100 µM ICG, (lower limit = 0.0) and to the DMSO control (upper limit = 1.0). Blue dots indicate the plate medians from three independent experiments, all control and chemical concentrations were tested in triplicates per plate. The black line indicates the best-fit concentrations response curve, the dotted line the corresponding 95% confidence interval. The blue dotted line indicates the BMC20, the concentration that inhibits the ^125^I-T4 uptake by 20%. The concentration range at which cytotoxicity was observed is indicated with a blue box. Compounds for which a control renormalization step was performed are marked with an^1^ (color figure online)

The 20 inhibitors from the CHO-K1 OATP1C1 range finding study were subsequently measured in eight concentrations to obtain full concentration–response curves (Fig. 3). TCS, BPA and BPAF all displayed sigmoidal concentration response patterns, but these responses were confounded by cytotoxic effects (Supplementary Figure S1). Cytotoxic effects were not observed for any other chemicals at the highest concentration tested (data not shown). LESI, PFOS, ICG, TBBPA, BSP and PCP displayed full sigmoidal concentration response patterns. Additionally, DIC and PFOA seem to display a sigmoidal concentration response pattern, but a full concentration response curve could not be established with these chemicals. BPZ, BPS, BPF, SY, VRP and RIF only inhibited T4 uptake at concentrations near the maximum concentration tested in the THTMT TH uptake assay and a full concentration response pattern could not be established. The concentrations response patterns for QCT and PRB appear to be more linear than sigmoidal, but no full concentration response pattern could be established. For each chemical, the BMC20 (i.e. 20% reduction in T4 uptake) was derived, which is provided together with the number of independent experiments, the lowest concentration with cytotoxic effect (IC10) and the best fit regression model in Table 2. TBBPA was the most potent OATP1C1 inhibitor (BMC20 = 0.0020 µM), followed by (in decreasing order of potency) QCT, PCP, ICG, PFOS, LESI, PRB, BSP, DIC, SR101, PFOA, SY, VRP, BPS, RIF and BPF. The concentration–response data observed for BPZ was considered as not sufficient for the derivation of a reliable BMC20 estimation. For three compounds (TCS, BPAF, and BPA) the BMC20s were in the range of cytotoxic concentrations, suggesting that the decrease in T4 uptake is not due to OATP1C1 inhibition, but rather due to reduced cell functioning.

**Table 2:** Inhibition of T4 uptake and cytotoxicity of individual compounds in the CHO-K1 OATP1C1 TH uptake assay

**Substance** **(by order of BMC20)**		Concentration response function					T4 uptake inhibition	Cytotoxicity
	N_study_	RM	${\widehat{\theta }}_{1}$	${\widehat{\theta }}_{2}$	${\widehat{\theta }}_{min}$	${\widehat{\theta }}_{max}$	BMC20 (M), [CI]	IC10 (M)
Tetrabromobisphenol A	4	Logit	− 23.30	− 2.82	0*	1.042	2.03E−09 [1.28E−09; 3.22E−09]	> 3.00E−07
Quercetin	3	Logit	− 3.90	− 0.73	0*	0.974	3.94E−08 [4.41E−09; 3.51E−07]	> 1.00E−04
Pentachlorophenol	4	Logit	− 8.05	− 1.35	0*	0.977	8.81E−08 [5.80E−08; 1.34E−07]	> 1.85E−05
Indocyanine green	4	Logit	− 13.10	− 2.19	0*	1.084	3.60E−07 [2.17E−07; 5.97E−07]	> 1.00E−04
Perfluorooctanesulfonic acid	3	Logit	− 10.23	− 1.84	0*	0.976	4.03E−07 [2.32E−07; 7.00E−07]	> 1.00E−04
Lesinurad	3	Logit	− 9.22	− 1.71	0*	0.954	4.34E−07 [1.97E−07; 9.56E−07]	> 1.00E−04
Probenecid	3	Logit	− 4.42	− 0.97	0*	0.962	6.66E−07 [1.24E−07; 3.57E−06]	> 1.00E−04
Bromosulfophthalein	4	Logit	− 11.37	− 2.16	0*	0.946	8.72E−07 [5.70E−07; 1.33E−06]	> 1.00E−04
Diclofenac	3	Logit	− 6.56	− 1.31	0*	1.062	1.40E−06 [6.84E−07; 2.87E−06]	> 1.00E−04
Triclosan	3	Logit	− 28.80	− 5.33	0*	1.025	2.30E−06 [1.38E−06; 3.83E−06]	3.78E−07
Sulforhodamine 101	4	Logit	− 13.60	− 2.69	0*	1.011	2.86E−06 [1.29E−06; 6.34E−06]	> 1.00E−05
Perfluorooctanoic acid	3	Logit	− 7.08	− 1.50	0*	1.036	2.94E−06 [1.04E−06; 8.36E−06]	> 1.00E−04
Bisphenol AF	4	Logit	− 32.69	− 6.27	0*	0.975	3.52E−06 [2.03E−06; 6.11E−06]	9.09E−07
Silychristin	3	Logit	− 14.50	− 2.92	0*	1.005	3.78E−06 [1.54E−06; 9.27E−06]	> 1.00E−05
Verapamil	3	Logit	− 7.60	− 1.88	0*	1.010	1.74E−05 [6.34E−06; 4.79E−05]	> 1.00E−04
Bisphenol A	4	Logit	− 14.60	− 3.35	0*	1.042	1.90E−05 [1.28E−05; 2.83E−05]	4.34E−06
Bisphenol S	3	Logit	− 7.72	− 2.07	0*	0.972	3.38E−05 [7.87E−06; 1.45E−04]	> 1.00E−04
Rifampicin	3	Logit	− 19.48	− 4.69	0*	1.063	4.06E−05 [2.58E−05; 6.38E−05]	> 1.00E−04
Bisphenol F	3	Logit	− 14.10	− 3.56	0*	1.004	4.49E−05 [2.80E−05; 7.20E−05]	> 1.00E−04
Bisphenol Z	4	Linear	–	− 2293	–	1.009	n.d. [n.d.; n.d.]	> 1.00E−04

BMC20: concentration that inhibits the T3 uptake by 20%; IC10: concentration that inhibits cell viability by 10% in the CellTiter-Glo® luminescent cell viability assay. Values in brackets denote the upper and lower limits of the approximate 95% confidence interval (CI); the column “RM” indicates the mathematical regression function as defined at Scholze et al. (2001): ${\widehat{\theta }}_{1},{\widehat{\theta }}_{2},{\widehat{\theta }}_{min},{\widehat{\theta }}_{max}$ estimated model parameters, given for concentrations expressed in M (rounded values), with * highlighting parameters that were not estimated but constrained to a fixed value. N_study_ = number of independent experiments

**Fig. 4:**
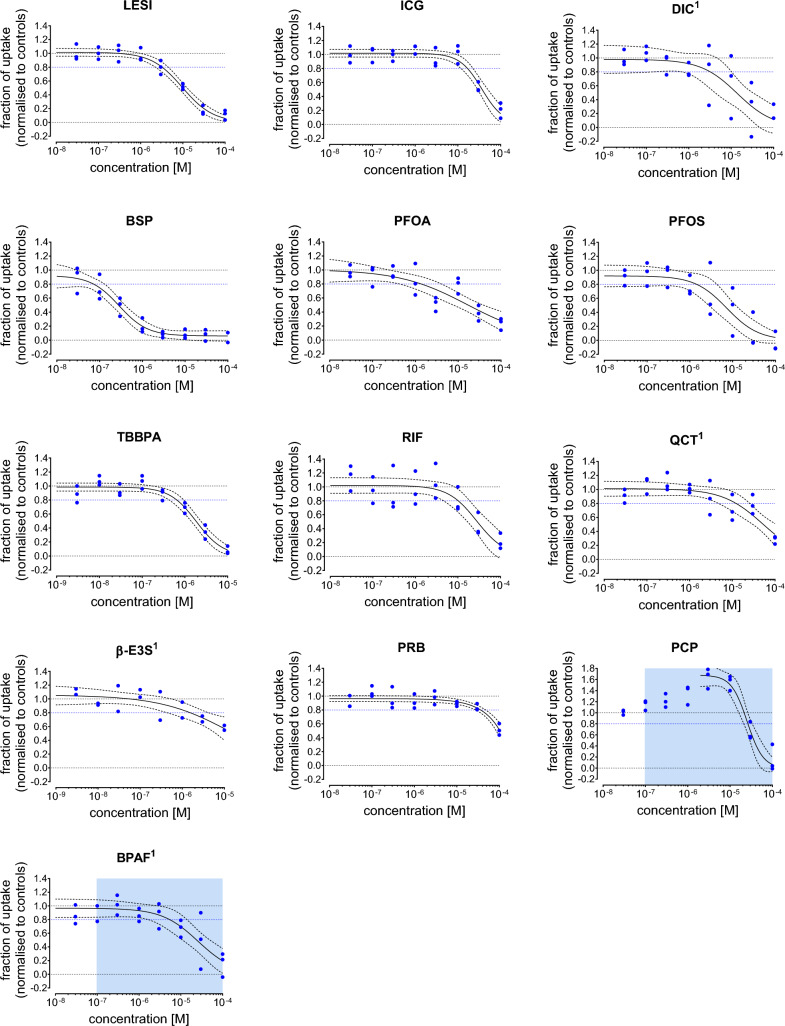
Concentration–response curves for test chemicals that showed OAT4 mediated uptake of T4 (10 nM) inhibition in the MDCK-OAT4 TH uptake assay. The fraction of ^125^I-T4 uptake is normalized to the positive control, 100 µM LESI (lower limit = 0.0) and to the DMSO control (upper limit = 1.0). Blue dots indicate the plate medians from three independent experiments, all control and chemical concentrations were tested in triplicates per plate. The black line indicates the best-fit concentrations response curve, the dotted line the corresponding 95% confidence interval. The blue dotted line indicates the BMC20, the concentration that inhibits the ^125^I-T4 uptake by 20%. The concentration range at which cytotoxicity was observed is indicated with a blue box. Compounds for which a control renormalization step was performed are marked with an^1^ (color figure online)

Thirteen inhibitors from the MDCK-OAT4 TH uptake range finding screening were also measured in eight concentrations to obtain full concentration response curves (Fig. 4). With the exception of PCP and BPAF (Supplementary Figure S2), none of the other chemicals were cytotoxic at the highest concentrations tested (data now shown). The BMC20, the number of independent experiments, the lowest concentration with cytotoxic effect and the best fit regression model used are summarized in Table 3. LESI, ICG, DIC, BSP, PFOS and TBBPA all displayed sigmoidal concentration response patterns. For PFOA, RIF, QCT and β-E3S a full concentration response pattern could not be established. BSP was the most potent OAT4 inhibitor (BMC20 = 0.072 µM), followed by TBBPA, PFOA, PFOS, β-E3S, DIC, LESI, RIF, QCT, ICG and PRB. The BMC20 values for BPAF and PCP were estimated at concentrations at which cytotoxic effects occurred, suggesting that the observed decrease in T4 uptake is due to reduced cell functioning, and not due to OAT4 inhibition specifically.

**Table 3:** Inhibition of uptake and cytotoxicity of individual compounds in the MDCK-OAT4 TH uptake assay

**Substance** **(by order of BMC20)**		Concentration response function					T4 uptake inhibition	Cytotoxicity
	N_study_	RM	${\widehat{\theta }}_{1}$	${\widehat{\theta }}_{2}$	${\widehat{\theta }}_{min}$	${\widehat{\theta }}_{max}$	BMC20 (M), [CI]	IC10 (M)
Bromosulfophthalein	3	Logit	− 19.76	− 3.02	0.059	0.922	7.25E−08 [3.85E−08; 1.36E−07]	> 1.00E−04
Tetrabromobisphenol A	3	Logit	− 18.37	− 3.21	0	0.986	6.49E−07 [4.32E−07; 9.74E−07]	> 1.00E−05
Perfluorooctanoic acid	3	Logit	− 5.98	− 1.25	0	1.006	1.38E−06 [4.27E−07; 4.44E−06]	> 1.00E−04
Perfluorooctanesulfonic acid	3	Logit	− 13.81	− 2.69	0	0.920	1.44E−06 [4.08E−07; 5.05E−06]	> 1.00E−04
ß-Estrone-3-sulfate	2	Logit	− 5.89	− 1.21	0	1.056	1.55E−06 [5.32E−07; 4.51E−06]	> 1.00E−05
Diclofenac	3	Logit	− 11.41	− 2.35	0	0.980	3.22E−06 [6.39E−07; 1.62E−05]	> 1.00E−04
Lesinurad	3	Logit	− 13.72	− 2.74	0	1.016	3.23E−06 [2.26E−06; 4.61E−06]	> 1.00E−04
Bisphenol AF	3	Logit	− 10.62	− 2.32	0	0.966	5.39E−06 [1.65E−06; 1.76E−05]	9.69E−08
Rifampicin	3	Logit	− 13.49	− 2.96	0	1.021	1.02E−05 [4.85E−06; 2.14E−05]	> 1.00E−04
Quercetin	3	Logit	− 9.12	− 2.13	0	1.010	1.23E−05 [4.77E−06; 3.16E−05]	> 1.00E−04
Indocyanine green	3	Logit	− 17.36	− 3.91	0	1.017	1.68E−05 [1.20E−05; 2.33E−05]	> 1.00E−05
Pentachlorophenol	3	Logit	− 24.58	− 5.35	0	1.678	2.65E−05 [2.17E−05; 3.23E−05]	1.14E−06
Probenecid	3	Linear	–	− 4474	0	0.963	3.65E−05 [2.69E−05; 4.61E−05]	> 1.00E−04

BMC20: concentration that inhibits the T3 uptake by 20%; IC10: concentration that inhibits cell viability by 10% in the CellTiter-Glo® luminescent cell viability assay. Values in brackets denote the upper and lower limits of the approximate 95% confidence interval (CI); the column “RM” indicates the mathematical regression function as defined at Scholze et al. (2001): ${\widehat{\theta }}_{1},{\widehat{\theta }}_{2},{\widehat{\theta }}_{min},{\widehat{\theta }}_{max}$ estimated model parameters, given for concentrations expressed in M (rounded values), with * highlighting parameters that were not estimated but set to a fixed value. N_study_ = number of independent experiments

## Discussion

In this study we developed in vitro TH uptake assays to screen for potential EDCs acting upon OATP1C1 and OAT4 facilitated transmembrane transport of TH. We validated both TH uptake assays by using negative controls and known inhibitors as positive control. Moreover we showed the inhibitory effects of a number of environmental contaminants. As expected, in both OATP1C1 and OAT4 overexpressing cell lines the uptake of ^125^I-T4 was higher than in the control cell lines. Several studies have tested the effect of chemicals on T4 transmembrane transport by rat, murine or human OATP1C1 in HEK293 transfected cells lines. Westholm et al. (2009) showed that several nonsteroidal anti-inflammatory drugs (NSAIDS), such as DIC, as well as ICG can inhibit T4 (1 nM) transmembrane transport by oatp1c1 with an IC50 of 4 µM and 0.1 µM, respectively. Tohyama et al. (2004) demonstrated that PRB is a weak inhibitor of oatp1c1 T4 facilitated transmembrane transport (Ki value = 293 µM). Bakos et al. (2020) found that the fluorescent dye SR101 was a substrate of OATP1C1 that could compete with T4 uptake. In our TH uptake assay, these compounds also interfered with OATP1C1-facilitated T4 uptake at similar potencies. Moreover, BCH and IBU were previously shown not to interact with OATP1C1, which was confirmed in our in vitro TH uptake assay. For OAT4, only PRB and LESI have been reported as OAT4 inhibitors interfering with T4 transmembrane transport (Chen et al. 2023). Both compounds also inhibited T4 uptake by OAT4 in our TH uptake assay. The high agreement between previously published data and our screening results confirms that our in vitro TH uptake assays can reliable detect chemicals known to be inhibitors of OATP1C1 or OAT4.

In addition to already known inhibitors of OATP1C1 and OAT4, we also tested various environmentally relevant industrial chemicals and pharmaceuticals. We demonstrated that TBBPA is a potent OATP1C1 inhibitor, while other bisphenols only exerted weak to no inhibition. TBBPA is considered as a widely discussed chemical with regards to TH-dependent endocrine disruption and neurotoxic effects: in rats, TBBPA is known to decrease serum (free) T4, while it simultaneously causes thinning of the brain cortex, but it exerts no effect on serum T3 or TSH (Cope et al. 2015). Several other studies have shown a similar effect of TBBPA on circulating T4 in both rat and mice (Sinkó et al. 2022; Van der Ven et al. 2008). Moreover, auditory deficits have been reported after TBBPA exposure in both rats and mice (Lilienthal et al. 2008; Park et al. 2016). A recent study has found that TBBPA exposure causes decreased TH concentrations and thyroid histological changes, but has no overt effect on brain development and neurological behavior in mice (Song et al. 2024). The risk of TBBPA in humans is still heavily debated, some studies suggests that, due its rapid metabolism and excretion in humans, TBBPA should not be considered a neurotoxicant for humans (Kacew and Hayes 2020). However, there are still concerns about the neurotoxic effect of TBBPA in embryos and continuously exposed humans (Dong et al. 2021; Feiteiro et al. 2021; Zhou et al. 2020). Limited data are available on the effect of TBBPA on TH concentrations and neurological effects in humans. A cross-sectional study in Belgium found no significant effect of TBBPA exposure on circulating TH concentrations or any neurobehavioral characteristics (Kiciński et al. 2012). TBBPA is a known potent transthyretin (TTR) binder, competing with T4 for binding to TTR (Hamers et al. 2006). Our data indicate a strong correlation between the potencies of chemicals displaying inhibited OATP1C1-mediated T4 transport and TTR binding. PCP is similarly a potent TTR binder (Hamers et al. 2006), which in our TH uptake assay significantly reduced T4 uptake by OATP1C1. In rat animal models, exposure to PCP significantly reduced the transport of T4 into the brain and CSF (Van Raaij et al. 1994), in ram lambs exposure to PCP was associated with a lower serum T4 concentration (Beard et al. 1999). In a Dutch cohort, PCP in umbilical cord blood has been associated with reduced serum TH concentrations as well as reduced neuropsychological functioning, including cognitive, motor function and behavioral performance, in 5–6 year old children (Roze et al. 2009). Similarly in a Canadian cohort, high serum and umbilical cord concentrations of PCP were associated with lower free T4 concentrations in newborns (Sandau et al. 2002). In our study, other known TTR inhibitors, such as PFOS and PFOA also reduced the uptake of T4 in both the OATP1C1 and OAT4 overexpressing cell lines. PFOS and PFOA are known substrates of OAT4 and are transported through OAT4 in placenta perfusion models (Kummu et al. 2015). Moreover, PFOS and PFOA can cross the BCSFB and accumulate in the CSF (Hu et al. 2023; Wang et al. 2018). In addition to PFOS and PFOA, many other perfluorinated chemicals are transported by OAT4, including PFDA, PFNA, PFHpA and PFHxS (Louisse et al. 2023).

The miniaturization of the THTMT TH uptake assays in 96 wells format allows for quick screening of multiple chemicals. Recently similar studies were published on MCT8 inhibition using a non-radioactive method (Johannes et al. 2016; Wagenaars et al. 2023). This method is based on the Sandell-Kolthoff methodology, which uses a colorimetric redox reaction of cerium and arsenate (i.e. the Sandell-Kolthoff reaction (Sandell and Kolthoff 1937)) to indirectly quantify uptake of THs by measuring the iodide in cells (Jayarama-Naidu et al. 2015; Renko et al. 2012). For HTS purposes, a non-radioactive method would be more preferable. However the non-radioactive method was not feasible with the current CHO-K1 OATP1C1 and MDCK-OAT4 TH uptake assay, most probably due to the sensitivity limitations of the non-radioactive method. Although the radioactive TH uptake assays is more sensitive, it also requires expensive radioactive isotopes that can only be used in a specialized lab and produces radioactive waste. Nonetheless, both methods are be suitable for the screening of possible THTMT inhibitors.

**Fig. 5:**
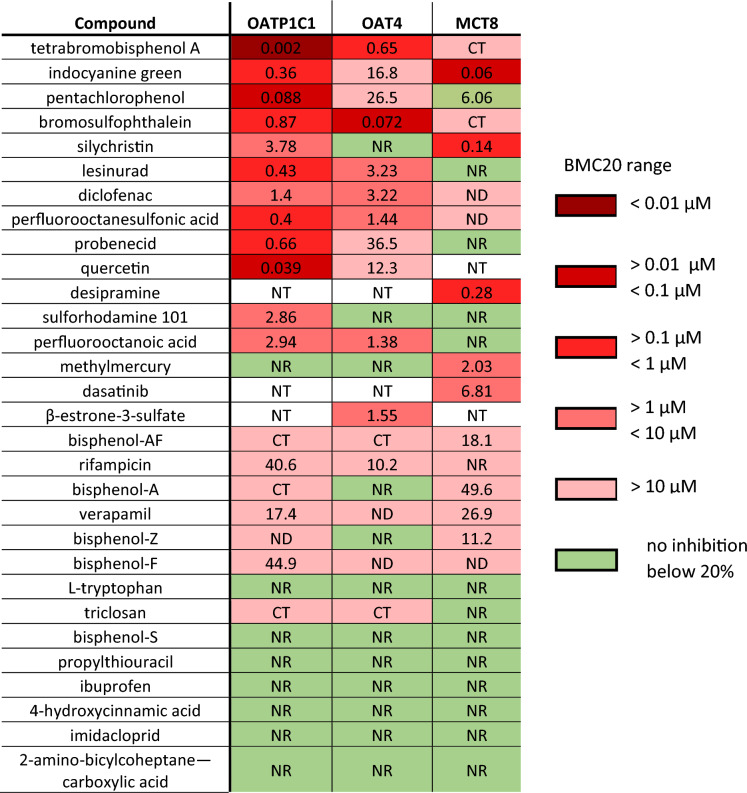
Overview of the potency of THTMT inhibitors on different THTMT TH uptake assays. Data for the MCT8 column was taken from Wagenaars et al. (2023). The calculated BMC20 value is given for each THTMT TH uptake assay, as well as a color graded range of BMC20 as shown in the figure legend. Compounds for which no inhibition below 20% was observed are marked in red. ND = no BMC20 value was determined, NR = no significant response observed, NT = not tested, CT = cytotoxic at BMC20 (color figure online)

A summary overview of the potencies of all tested chemicals in each THTMT TH uptake assay, as well as data from our previous study on MCT8 inhibition (Wagenaars et al. 2023) is presented in Fig. 5. A large overlap in inhibitory effect on TH uptake exists between the tested chemicals on each THTMT, although the potencies of each inhibitor can differ greatly between THTMTs. For example, TBBPA, PCP and QCT have very high inhibitory potencies in the CHO-K1 OATP1C1 TH uptake assay but > tenfold lower values in the MDCK-OAT4 and MDCK-MCT8 TH uptake assay. Similarly, SY and ICG appear to be very potent in the MDCK-MCT8 TH uptake assay, but with a much lower potency in the CHO-K1 OATP1C1 and MDCK-OAT4 TH uptake assay. BSP is a very potent OAT4 inhibitor, with a lower potency in the CHO-K1 OATP1C1 and MDCK-MCT8 TH uptake assay. These data show that THTMT TH uptake assays are suitable to identify THTMT specific inhibitors and might be vital in future studies to elucidate the contribution of THTMTs on the total TH uptake. Moreover, a more extensive screening of chemicals in a battery of THTMT overexpressing cell lines assays can help to identify chemicals which inhibit multiple THTMTs and are more likely to cause adversity in vivo. A battery of in vitro THTMTs TH uptake assays should therefore include the most important THTMTs identified so far as well as any newly identified important THTMTs.

For many of the chemicals tested positively in our THTMT TH uptake assays, TH system disrupting effects have been reported in animal toxicity studies or in human epidemiological studies. These studies usually only report TH changes in blood serum concentrations. THTMT inhibition, however, may not always be reflected by changes in serum TH concentrations, as shown by OATP1C1 knock out models and the OATP1C1 deficient patient (Mayerl et al. 2012; Strømme et al. 2018). Therefore, unless structural neurological and neurobehavioral effects are described, as in the case for PFOS, PFOA, PCP and TBBPA (Roze et al. 2009), the interpretation of our data in relation to in vivo effects becomes more complicated. The lack of in vivo animal studies reporting TH concentrations in brain most likely stems from the analytical difficulties of measuring TH in a complex matrix such as brain homogenates. However, recent efforts have improved TH analytics of rodent and mouse brain (De Angelis et al. 2022; Ford et al. 2023). As such TH measurements of brain content will become more prominent in the future and can give more insights into the effect of THTMT inhibitors on brain TH content in vivo. Only few studies have reported on the effect of EDCs on the transport of TH into the cerebrospinal fluid (CSF), even though the CSF has been acknowledged as an important reservoir for TH (Dratman et al. 1991; Richardson et al. 2015). TH concentrations in CSF and TH CSF:serum ratios can be indicative of neurological adverse effects, even when serum TH concentrations are within normal ranges (Funkquist et al. 2020). Thus, incorporating CSF sampling in in vivo animal studies on EDCs would provide important data with regards to the effect of THTMT inhibitors, especially for THTMTs present in the BCSFB.

In conclusion, we developed two in vitro assays for the detection of EDCs acting upon OATP1C1 and OAT4 facilitated transmembrane transport of T4. Both THTMT TH uptake assays corresponded well with previously available literature. Furthermore, we identified several chemicals that inhibited OATP1C1 facilitated T4 transmembrane transport, including TBBPA, PCP, PFOS and PFOA. Additionally, we identified BSP, TBBPA, PFOS and PFOA as a potent inhibitor of OAT4 mediated transport. These THTMT TH uptake assays can be used to rapidly screen for potential EDCs interfering with TH transport across physiological barriers, which can provide more information on the molecular mechanisms of TH system disrupting chemicals. Moreover, data from these THTMT TH uptake assays can be used to prioritize EDCs for further toxicological studies.

### Supplementary Information

Below is the link to the electronic supplementary material.

Supplementary file1 (DOCX 284 KB)

## Data Availability

The data that support the findings of this study are available from the corresponding author, upon reasonable request.

## Abbreviations

4HCA: 4-Hydroxycinnamic acid
AHDS: Allan–Herndon–Dudley syndrome
BBB: Blood–brain-barrier
BCH: 2-Amino-bicycloheptane-2-carboxylic acid
BCSFB: Blood–cerebral-spinal-fluid barrier
β-E3S: β-Estradiol-3-sulfate
BMC: Benchmark concentration
BMR: Benchmark response level
BPA: Bisphenol-A
BPAF: Bisphenol-AF
BPF: Bisphenol-F
BPS: Bisphenol-S
BPZ: Bisphenol-Z
BSP: Bromosulfopthalein
BSA: Bovine serum albumin
CHO-K1: Chinese hamster ovary
cpm: Counts per minute
DIC: Diclofenac
DIO: Deiodinase
DMI: Desipramine
DMSO: Dimethylsulfoxide
EDCs: Endocrine disrupting chemicals
HTS: High-throughput screening
IBU: Ibuprofen
IC: Inhibitory concentration
ICG: Indocyanine green
IMI: Imidacloprid
LESI: Lesinurad
MCT8: Monocarboxylate transporter 8
MDCK: Madin Darby canine kidney
MeHg: Methyl mercury
NaOH: Sodium hydroxide
NSAIDS: Nonsteroidal anti-inflammatory drugs
OATP1C1: Organic anion transporter polypeptide 1C1 (human)
Oatp1c1: Organic anion transporter polypeptide 1C1 (rodent)
OAT4: Organic anion transporter 4
PBS: Phosphate-buffered saline
PCP: Pentachlorophenol
PFOA: Perfluorooctanoic acid
PFOS: Perfluorooctane sulfonic acid
PRB: Probenecid
PTU: Propylthiouracil
QCT: Quercetin
RIF: Rifampicin
rT3: Reverse T3
SR101: Sulforhodamine 101
SY: Silychristin
T3: 3,5,3′-Tri-iodothyronine
T4: Thyroxine
TBBPA: Tetrabromobisphenol-A
TBBPS: Tetrabromobisphenol-S
TCS: Triclosan
TH: Thyroid hormone
THS: Thyroid hormone system
THTMT: Thyroid hormone transmembrane transporter
TRP: l-Tryptophan
TTR: Transthyretin
UA: Uric acid
VRP: Verapamil
WT: Wildtype
