# Supplementary material for: Two novel in vitro assays to screen chemicals for their capacity to inhibit thyroid hormone transmembrane transporter proteins OATP1C1 and OAT4
Source: Arch Toxicol. 2024 May 18;98(9):3019–34. doi: 10.1007/s00204-024-03787-2 (PMC11324666; doi:10.1007/s00204-024-03787-2)
Supplement: Supplementary file 1 — Supplementary file1 (DOCX 284 KB) [file 204_2024_3787_MOESM1_ESM.docx]

**Supplementary material**

Figure S1. Concentration-response curves of the CellTiter-Glo® luminescent cell viability assay for test chemicals that exerted cytotoxic effects on CHO-K1 OATP1C1 cells. Cell viability is normalized to the positive control (lower limit = 0.0) and to the DMSO control (upper limit = 1.0). Blue dots indicate the plate medians from three or four independent experiments, all control and chemical concentrations were tested in triplicates per plate. The black line indicates the best-fit concentrations response curve, the dotted line the corresponding 95% confidence interval. The blue dotted line indicates the IC10, the concentration that decreased cell viability by 10%.

Figure S2. Concentration-response curves of the CellTiter-Glo® luminescent cell viability assay for test chemicals that exerted cytotoxic effects on MDCK-OAT4 cells. Cell viability is normalized to the positive control (lower limit = 0.0) and to the DMSO control (upper limit = 1.0). Blue dots indicate the plate medians from three or four independent experiments, all control and chemical concentrations were tested in triplicates per plate. The black line indicates the best-fit concentrations response curve, the dotted line the corresponding 95% confidence interval. The blue dotted line indicates the IC10, the concentration that decreased cell viability by 10%.
